# Trefoil System for the Treatment of Mandibular Edentulism: A Case Report with 30 Months Follow-Up

**DOI:** 10.1155/2020/8845649

**Published:** 2020-10-17

**Authors:** Andrea E. Borgonovo, Simone L. M. Galbiati, Dino Re

**Affiliations:** Department of Aesthetic Dentistry, University of Milan, Istituto Stomatologico Italiano, Milan, Italy

## Abstract

The purpose of this work is to describe a clinical case of full-arch mandible rehabilitation with a fixed prosthesis on three implants. The chosen protocol is the Trefoil system by Nobel (Nobel Biocare, Zurich, Switzerland) that allows to realize a mandibular fixed rehabilitation on three particularly designed fixtures through the use of prefabricated surgical guides and a preassembled bar on which the prosthesis is built. Both surgical and prosthetic procedures were completed without complications, and after 30 months, the rehabilitations are in good health conditions. The patient is able to maintain a good level of hygiene and is satisfied with the work from an aesthetic and functional point of view.

## 1. Introduction

After tooth loss, which occurs more often in senior people, when the rehabilitation involves a fixed prosthesis on implants, the ideal solution would be alveolar ridge regeneration [1–4]. This kind of surgery is aimed at restoring the correct bone anatomy and allows the insertion of more implants, particularly in the molar region, thus creating a prosthesis without distal cantilever and orthopedic resin gingiva. The survival rate of dental implants is high, regardless of the bone augmentation technique chosen, but such procedures require great operator skills and a long healing period [5, 6]. Moreover, they imply high morbidity and treatment costs, and they need strong patient compliance to achieve clinical success.

The economic aspect of the rehabilitation often guides patients in their choice and, when it is possible, they prefer fixed rehabilitations instead of removable. The aim of modern dentistry is, therefore, to develop minimally invasive rehabilitations that ensure function, aesthetics, comfort, and cost containment.

In the case of edentulous mandible, the Branemark protocol with five axial fixtures between the two mandibular foramina has undergone several changes over the years. Immediate loading has almost routinely replaced conventional loading protocols with long-term predictability and success rate [7, 8]. Malò and Rangert introduced the greatest innovation regarding the number of implants thanks to his all-on-four concept. This technique consists of the insertion of two axial implants in the lateral incisor/canine area and two fixtures just anterior to the foramina intentionally tilted distally about 30° relative to the occlusal plane which thus merging at the second premolar position. This arrangement allows for good implant anchorage, short cantilever length, and large interimplant distance [9]; moreover, it can also be used with six implants.

The implants number is further decreased in the Branemark Novum Concept with only three fixtures. This protocol involves prefabricated components, elimination of the impression procedure, and attachment of the permanent fixed bridge on the day of implant placement [10–12].

The Trefoil system by Nobel Biocare (Nobel Biocare, Zurich, Switzerland) is an evolution of the Branemark Novum Concept. Three particularly designed fixtures are placed in the anterior part of the mandible using drilling templates and then immediately splinted with a prefabricated titanium bar. This bar simplifies the creation of the final acrylic prosthesis and saves time for both the clinician and the laboratory. It is anatomically designed for the natural curvature of the mandible, it features adaptable joints that adjust to compensate for horizontal, and vertical and angular deviations from the ideal implant position and allow passive fit of the final prosthesis. The aim of this work is to show a clinical case realized with this technique.

## 2. Case Presentation

**Figure 1:**
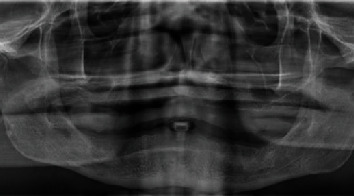
Preoperatory panoramic radiograph.

A male patient aged 65 and in good health conditions required a complete rehabilitation of the jaw. As it can be observed in the orthopantomography (Figure 1), he was completely edentulous and used two conventional removable prosthesis. It was decided to rehabilitate his lower jaw with Nobel Trefoil system surgical protocol.

After locoregional anesthesia into the vestibular and lingual mucosa of the mandible, a crestal incision connecting the two first molar regions was performed, and the alveolar ridge was exposed. The lingual flap was then sutured with a 2/0 silk suture in order to facilitate surgical proceedings and improve visibility.

**Figure 2:**
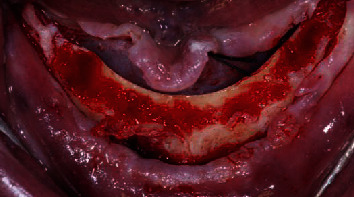
The alveolar ridge after the osteotomy: the flat surface is necessary for the application of the surgical templates.

Once the vestibular and the lingual mucosa had been protected with retractors, the surgeon regularized the alveolar ridge with an osteotomy drill. The aim of this step is to obtain a flat surface and an adequate thickness on the ridge for the application of the surgical guides and the following implant insertion (Figure 2).

**Figure 3:**
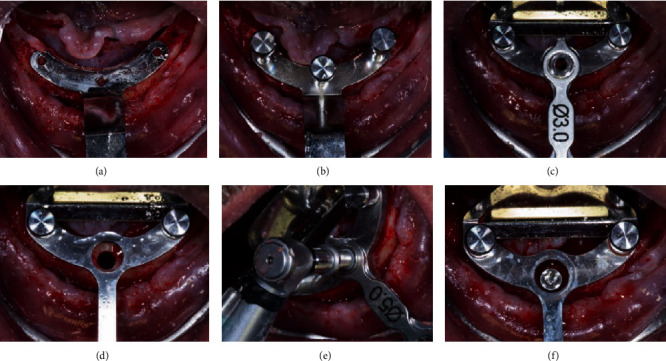
Intraoral view of the surgical steps (1): the first surgical guide is placed (a) and fixed (b), the central implant site is prepared thanks to metal bushings of increasing diameter (c, d), and the fixture is inserted (e, f).

With a pilot drill, the surgeon performed a guide bore in the center of the mandible and verified the correct orientation in occlusion with a parallelism pin. After that, the first surgical guide was positioned and fixed with a pin in the cavity previously drilled. The same guide has two other bores where the two distal implants will be placed. In these positions with the pilot drill, two new guide perforations were performed and used to fix the surgical guide. The first pin was thence removed and with the help of metal bushings of increasing diameter (from 3.00 mm to 5.00) mounted in the center bore on the guide the operator prepared the first implant site and inserted the fixture (Nobel Trefoil Implant 5.00 × 13 mm, Nobel Biocare, Zurich, Switzerland) (Figure 3).

**Figure 4:**
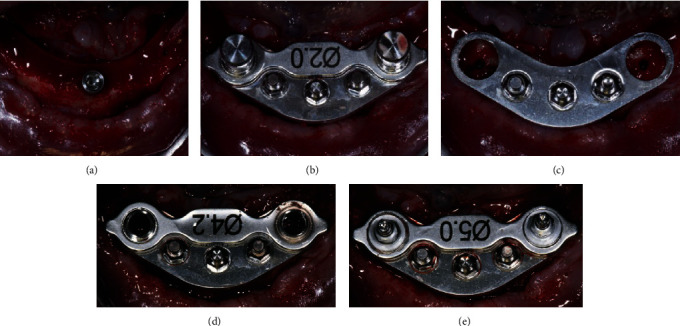
Intraoral view of the surgical steps (2): the first surgical guide is removed (a) and the second is placed and fixed (b, c), the distal implants sites are prepared thanks to metal bushings of increasing diameter (d), and the fixtures are inserted (e).

The surgical guide was removed and a new one was initially fixed on the implant. This second guide differs from the first because of the presence of two further bores next to the central one which is used to lock the guide and prevent it from rotating. Once this guide was completely locked even with appropriate pins in the cavities described, the bores in correspondence of the distal implants were, therefore, free and ready for the site preparation. A surgical template with metal bushings on its ends, different from that used for the first implant, was applied on the surgical guide and used to prepare the distal implants sites (Figure 4).

**Figure 5:**
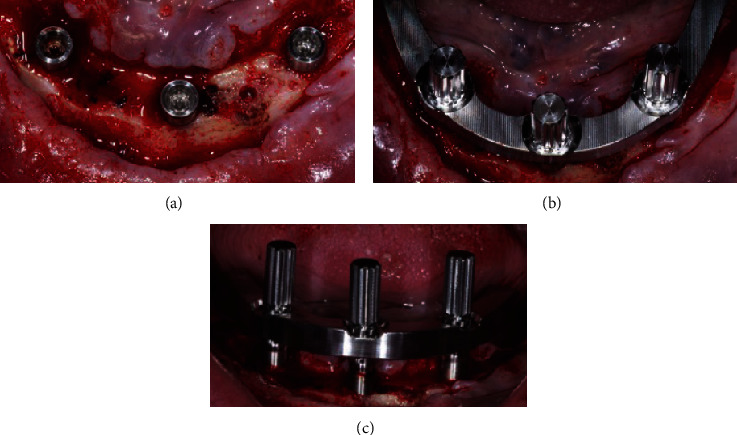
All the implants are placed (a), and the prefabricated titanium bar is screwed on the fixtures (b, c).

When the insertion of the distal fixtures (Nobel Trefoil Implant 5.00 × 13 mm, Nobel Biocare, Zurich, Switzerland) was completed even, the second guide was removed, and the prefabricated titanium bar was screwed onto the implants to check its fitting (Figure 5).

**Figure 6:**
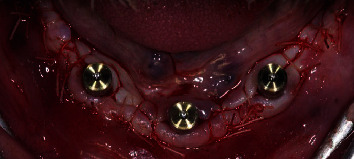
The surgical flap is sutured around the healing caps.

Next, the bar was substituted with the healing caps so that the surgeon could suture the flap around them with 4/0 resorbable thread (Figure 6).

**Figure 7:**
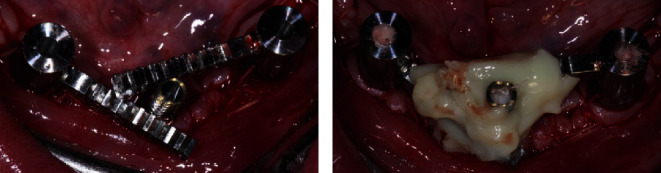
Three particularly designed transfers are screwed on the implants and splinted with composite.

As a last step, the transfers were placed on the implants and splinted together with composite in order to give the technician the right position of the fixture to build the prosthesis on the titanium bar (Figure 7).

**Figure 8:**
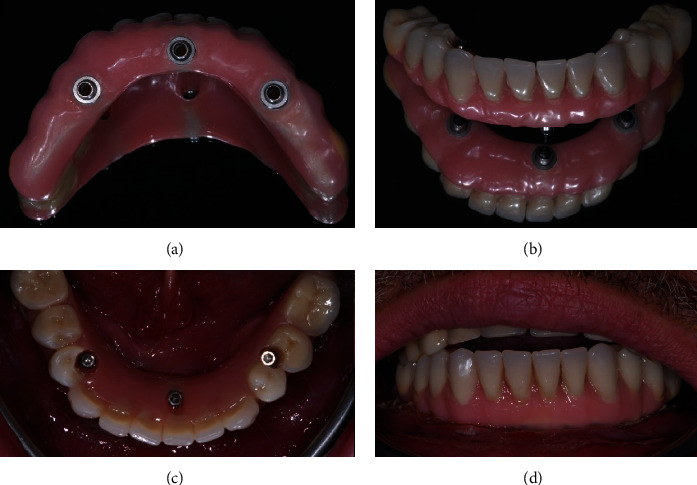
Extraoral view of the prosthesis before its insertion (a, b) and intraoral occlusal (c) and buccal (d) view once it has been screwed.

About four hours after the surgery, the prosthesis was screwed on the implants and functionalized in occlusion (Figure 8).

**Figure 9:**
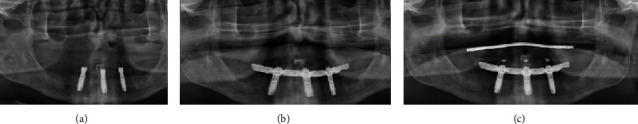
The OPT was taken immediately after the surgery (a), at six months (b), and during the last control visit (30 months).

The patient underwent a follow-up program and orthopantomographies were taken at six, eighteen, and thirty months after the surgery (Figure 9). The fixtures and the prosthesis appeared in good health both clinically and radiographically during the last control visit.

## 3. Discussion

The aim of reducing the number of implants in full arch fixed rehabilitations is to avoid complex and invasive surgeries such as bone augmentation and/or gingival grafts, improve hygiene maintenance, and reduce costs [13].

According to some reviews, full-arch fixed dental prosthesis in mandible supported by 2 to 4 implants exhibit a low rate of failures for implants and prostheses, a low rate of MBL, and a low rate of biomechanical and biological complications. One of these works, particularly, states that fixed prosthesis on three implants, when analyzed separately, has the lowest cumulative survival rates compared to the other group even if it is anyway high (95.5%). No statistically significant difference has been found in outcomes (implant and prosthesis survival) for full-arch FDPs in the mandible supported by less than five implants when compared to five or more implants. However, using 4–6 implants is a well-documented treatment option with high estimated 5-year survival of the construction, whereas it is unclear whether three implants for supporting a FAFDP will achieve similar survival rates [4, 13, 14].

The use of only three fixtures to support a full arch rehabilitation is not yet, therefore, confirmed in literature with specific systematic reviews or meta-analysis. Preliminary results of a recent study suggest that immediately loaded cross-arch prostheses can be supported by only three dental implants at least up to 1-year postloading but the Authors stated that longer follow-ups are needed to properly evaluate this therapeutic option [15].

Other works about all-on-three implants rehabilitation, like Branemark Novum Concept protocol, of which Trefoil represents the evolution, reported promising and encouraging outcomes with high survival rates both for implants and prosthesis but immediate loading seemed to be associated with lower survival of individual fixtures [10, 11]. A retrospective study with a 5-year follow-up about three implants (all-on-three) supporting a delayed loaded fixed prosthesis, indeed, showed that no implants were lost, giving a 100% success rate. The Authors stated that within the limits of the small group clinical study (24 patients and 72 implants), the high survival rate of the all-on-three protocol with delayed loading may be a viable concept [16].

A recent study with a long follow-up period (16 years) demonstrated very good long-term outcomes for the Branemark Novum protocol. No implant failed (CSR 100%) and no prosthesis needed to be substituted (CSR 100%). Only one biologic complication was detected on a central implant (crater-form bone destruction), and several prosthodontic complications occurred during the 16 years (fractures of resin or teeth), the majority of which were registered on the same parafunctional patient [12].

In the Authors' opinion, the Trefoil system is a valid therapeutic solution for the treatment of mandibular edentulism not only from the economic point of view. Fewer implants together with prefabricated components help to reduce cost and also to save time. The estimate of the active working time is in fact about six hours from the beginning of the surgery to the screwing of the prosthesis on the same day. It should be emphasized that the protocol suggested by Nobel provides for the immediate realization of the final prosthesis while usually in full arch rehabilitation cases a provisional one is needed. This leads to time-saving in the treatment plan of about 3-6 months.

On the other hand, the surgical protocol is not as simple as it may appear and the clinician must be well trained in order to manage any complication during the surgery such as proximity to the mental foramen which can lead to an intraoperative change of strategy. Moreover, it seems to be indicated for specific cases with a particular mandibular shape not very widespread in Italy and Europe but, particularly, in South America. At last, similar rehabilitations can be realized nowadays thanks to different technologies at our disposal. Digital, for example, allows us to plan the intervention and realize the prosthesis even before implant insertion with good reliability and similar savings in costs and time but with less complexity in the surgical phase.

## 4. Conclusion

In the realization of this particular clinical case, the Trefoil system proved to be a good surgical protocol and, after 30 months follow-up, the rehabilitation appeared in good health both for fixtures and the prosthesis. Moreover, the patient was satisfied with the work from an aesthetic, functional, and hygienic maintenance point of view.

In our opinion, this protocol can be useful in edentulous patients who present not only economic needs in the treatment plan but also short realization times. Both requests are achieved thanks to the use of prefabricated components.

Nevertheless, a wider number of cases with a long follow-up period are needed to provide a more correct assessment of the protocol.
